# YjbH regulates virulence genes expression and oxidative stress resistance in *Staphylococcus aureus*

**DOI:** 10.1080/21505594.2021.1875683

**Published:** 2021-01-25

**Authors:** Atmika Paudel, Suresh Panthee, Hiroshi Hamamoto, Tom Grunert, Kazuhisa Sekimizu

**Affiliations:** a Teikyo University Institute of Medical Mycology, Hachioji, Tokyo, Japan; b Division of Infection and Immunity, Research Center for Zoonosis Control, Hokkaido University, Sapporo, Japan; c Functional Microbiology, Institute of Microbiology, Department of Pathobiology, University of Veterinary Medicine, Vienna, Austria

**Keywords:** *Staphylococcus aureus*, virulence, YjbH, YjbI, oxidative stress, surface structure, gene expression, protease, pigment, silkworm

## Abstract

We previously reported that disruption of the *yjbI* gene reduced virulence of *Staphylococcus aureus*. In this study, we found virulence in both silkworms and mice was restored by introducing the *yjbH* gene but not the *yjbI* gene to both *yjbI* and *yjbH* genes-disrupted mutants, suggesting that *yjbH*, the gene downstream to the *yjbI* gene in a two-gene operon-*yjbIH*, is responsible for this phenomenon. We further observed a decrease in various surface-associated proteins and changes in cell envelope glycostructures in the mutants. RNA-seq analysis revealed that disruption of the *yjbI* and the *yjbH* genes resulted in differential expression of a broad range of genes, notably, significant downregulation of genes involved in virulence and oxidative stress. Administration of N-acetyl-L-cysteine, a free-radical scavenger, restored the virulence in both the mutants. Our findings suggested that YjbH plays a role in staphylococcal pathogenicity by regulating virulence gene expression, affecting the bacterial surface structure, and conferring resistance to oxidative stress in a host.

## Introduction

*Staphylococcus aureus* expresses a plethora of virulence factors to invade, thrive, and multiply within the host 1,2. As a human-commensal and an opportunistic pathogen, it is responsible for several infectious diseases ranging from minor skin infections to life-threatening endocarditis [3]. Moreover, the spread of multi-drug resistant *S. aureus* strains has challenged the treatments, thus warranting novel therapeutic options [4–10]. It is crucial to understand the pathogen, its weapons, and host-pathogen interactions to develop novel therapeutics. One way of combating infections is to disarm the pathogen from its virulence factors, rendering it less pathogenic, which helps the host immunity expel the pathogen quickly. In this sense, exploring the mechanism of virulence regulation will enhance the understanding of pathogenesis and host-pathogen interaction. To identify the *S. aureus* virulence factors and the genes responsible for pathogenicity, we performed screening using a silkworm infection model. Over the past years, we have identified several *S. aureus* virulence factors that are involved in both the silkworm and mice virulence [11–14].

Recently, from the screening of 380 transposon insertion mutants of *S. aureus* USA300 of Nebraska Transposon Mutant Library, we found that disruption of the *yjbI* gene, lying upstream of the *yjbH* gene in a two-gene operon, resulted in the loss of pathogenicity in silkworm and mice. Pathogenicity was restored by the introduction of the *yjbIH* operon and not of the *yjbI* gene [14], suggesting that the *yjbH* gene might have a role in pathogenicity. Studies involving YjbH in *Bacillus subtilis* [15–17], *S. aureus* [18–21], and other Gram-positive bacteria [22–24] have reported it to be an adaptor protein responsible for proteolysis of Spx, a transcriptional regulator, via ClpXP protease [16–18,24–26]. However, the role of YjbH in the pathogenicity of *S. aureus* is obscure. In this study, we found that YjbH is essential to *S. aureus* virulence in a whole-body animal infection model and involved in the expression of the broad range of virulence genes. In addition, we found that YjbH affects the bacterial surface structure, and confers resistance to oxidative stress in the host.

## Materials and methods

### Bacterial strains and culture conditions

Bacterial strains used in this study are summarized in Table 1. *S. aureus* strains were grown in tryptic-soy broth (TSB; Becton Dickinson and Company, Franklin Lakes, NJ, USA). *Escherichia coli* was grown in Luria-Bertani medium (tryptone 10 g/l, yeast extract 5 g/l, NaCl 10 g/l, pH 7.0). Antibiotics were supplemented as required.

**Table 1.** Bacteria used in this study

Bacteria	Description	Source
*Staphylococcus aureus* JE2	Plasmid-cured derivative of USA300 CA-MRSA strain LAC, parent strain for *bursa aurealis* transposon insertion.	[32]
*S. aureus yjbI*::Tn	Strain derived from JE2 with transposon inserted in the *yjbI* (*SAUSA300_0904*) gene	[32]
*S. aureus yjbH*::Tn	Strain derived from JE2 with transposon inserted in the *yjbH* (*SAUSA300_0903*) gene	[32]
*S. aureus* RN4220	Restriction deficient strain derived from *S. aureus* NCTC8325-4	[59]
*S. aureus yjbI*::Tnp*yjbI*	*S. aureus yjbI*::Tn harboring intact *yjbI* gene with plasmid pSR515	[14]
*S. aureus yjbI*::Tnp*yjbIH*	*S. aureus yjbI*::Tn harboring intact *yjbIH* operon with plasmid pSR515	[14]
*S. aureus yjbH*::Tnp*yjbI*	*S. aureus yjbH*::Tn harboring intact *yjbI* gene with plasmid pSR515	This study
*S. aureus yjbH*::Tnp*yjbIH*	*S. aureus yjbH*::Tn harboring intact *yjbIH* operon with plasmid pSR515	This study
*S. aureus yjbH*::Tnp*yjbH*	*S. aureus yjbH*::Tn harboring intact *yjbH* gene with plasmid pND50pfbaA	This study
*Escherichia coli* HST08	General purpose host strain for cloning	Takara Bio

### Silkworm rearing and infection assay

Silkworms rearing until the fourth molt stage was performed on antibiotic-containing artificial diet Silkmate 2S (Nihon Nosan Corp., Japan) as previously described [27]. The fifth instar larvae were fed on an antibiotic-free artificial diet (Sysmex, Japan) overnight and injected with 50 µl of bacterial strains into the hemolymph (n = 5). The silkworms were kept at 27°C incubator, and survival was recorded.

### Pathogenicity in mice

Mice experiments were performed with approval from Teikyo University Animal Ethics Committee (18–024). Bacterial strains were cultured overnight in TSB with or without antibiotics. Erythromycin (5 µg/ml) alone or with chloramphenicol (12.5 µg/ml) was added to the medium for transposon mutants or gene complemented strains, respectively. The overnight cultures were 100-fold diluted with TSB and continued incubation for 16 h at 37°C with shaking. The cells were collected by centrifugation, washed with phosphate-buffered saline (PBS), and suspended in PBS to adjust the turbidity (A_600_) to 0.7 for microbial load and 4.0 for survival assay. The prepared suspensions (200 µl) of the strains were injected into the tail-vein of mice (ICR, female, eight weeks old, Charles River Laboratories, Kanagawa, Japan). The CFU of the injected suspensions was counted. For measuring the microbial load, kidney and heart of the infected mice were isolated after 24 h of injection, suspended in PBS, homogenized, and CFU was counted.

### Pigment production and proteolysis

Bacteria were grown overnight in TSB medium at 37°C with shaking. Two microliters of the overnight cultures were spotted on TSB agar plates alone or supplemented with 3.3% skim milk to determine pigment production and proteolysis, respectively, and incubated at 37°C overnight. Pigment production was assessed visually by the color of the colony formed on TSB agar plates after overnight incubation. The proteolytic activity was determined by the appearance of a clear zone surrounding the bacterial colonies.

### Plasmid construction and complementation

The *yjbH* gene was amplified using primers SAUSA300_0903_Bam_F: CGCGGATCCATGGCT GGAGAATTACGAATAAT and SAUSA300_0903_Sal_R: ACGCGTCGACTTATTTTGATTTGATTTTAGGCATT, ligated to the BamHI/SalI-digestedpND50-pfbaA vector [14] and transformed into *E. coli* HST08. Bacteriophage 80α-mediated complementation of the plasmids was performed into the respective mutants as previously described [14].

### RNA-Sequencing and analysis

The overnight culture of the strains was diluted 100-fold and incubated at 37°C with aeration until A_600_ was 1.0. RNA stabilization, extraction, and sequencing were performed, as explained in the previous report [28]. Differentially expressed genes were identified using CLC Genomics Workbench software, version 12.0 (CLC Bio, Aarhus, Denmark). Reads were aligned to the USA300 genome allowing a minimum length fraction of 0.95 and minimum similarity fraction of 0.95. Genes with false discovery rate (FDR) *p* < 0.05 and minimum fold expression 2 were classified as having significantly different expression. Functional analysis of differentially expressed genes was performed using GO enrichment analysis, as explained previously [29,30].

### Surface protein profiling

Strains were grown overnight in TSB or TSB supplemented with antibiotic as necessary at 37°C with aeration, and the A_600_ of the full growth was adjusted to 9.0. Five ml of OD adjusted overnight culture was centrifuged to collect the cells. The pellets were washed with PBS and suspended in sodium dodecyl sulfate-polyacrylamide gel electrophoresis (SDS-PAGE) sample buffer and centrifuged. The supernatant was then boiled for 5 minutes, and 20 µl was loaded to a 4–20% gradient Mini-PROTEAN®TGX™ precast gel (Bio-Rad Laboratories, Inc., Hercules, CA, USA). After electrophoresis, the gel was stained with Coomassie brilliant blue for visualization.

### Fourier-Transform Infrared spectroscopic analysis

Fourier-Transform Infrared (FTIR) spectroscopic measurements and spectral evaluation were performed as previously reported [31]. Briefly, wild-type, *yjbI::Tn*, and *yjbH::Tn* strains were grown at TSA, 37°C for 24 h and were subsequently analyzed by whole-cell FTIR spectroscopy. Measurements were conducted on a Tensor 27/HTS-XT microplate adapter FTIR spectrometer (Bruker Optics GmbH, Ettlingen, Germany). Unsupervised hierarchical cluster analysis (HCA, average linkage clustering) was performed on the second derivative, and vector normalized spectra using the highly discriminatory spectral region between 1200–800 cm^−1^, which is dominated by vibrations of various oligo- and poly-saccharides and their specific type of glycosidic linkages.

## Results

### *The* yjbH *gene is responsible for the pathogenicity of* S. aureus

**Figure 1.**
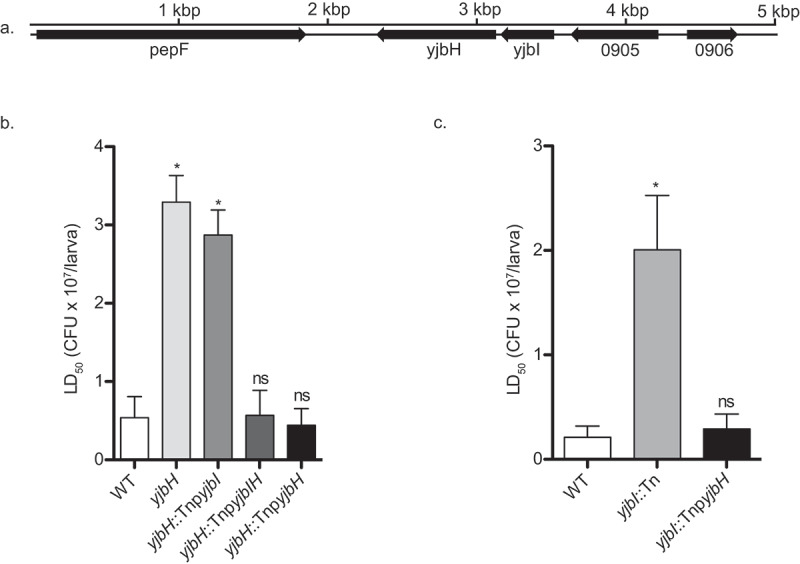
The *yjbH* gene is responsible for pathogenicity in silkworm. (a). Gene organization. (b). Pathogenicity of *yjbH*::Tn introduced with the *yjbI, yjbIH, and yjbH* genes c. Pathogenicity of *yjbI*::Tn strain introduced with the *yjbH* gene. Overnight cultures of *S. aureus* strains were serially diluted and injected into the silkworm hemolymph (n = 5), and LD_50_ values were calculated from the survival at 30 h post-infection by logistic regression analysis using logit link function. Data are mean ± SEM of triplicate experiments and analyzed by one-way analysis of variance (ANOVA) with Dunnett’s multiple comparison test compared with the WT (* *p* < 0.05)

Recently, we identified *yjbI*::Tn to have reduced pathogenicity, pigment, and protease productions [14]. We also reported that the introduction of the *yjbI* gene did not restore pathogenicity in *yjbI*::Tn, while the introduction of the operon consisting of the *yjbI* gene and the downstream gene *yjbH* (Figure 1(a)) restored the pathogenicity [14]. This result, together with a previous report where survival of Δ*yjbH* in whole blood assay was reduced [19], indicated that YjbH might have roles in pathogenicity. Therefore, we examined the role of the *yjbH* gene in pathogenicity to silkworms. We observed that the *yjbH*::Tn mutant had reduced silkworms-killing ability (Figure 1(b)), and the pathogenicity to silkworms was restored when the *yjbIH* operon or only the *yjbH* gene were introduced to *yjbH*::Tn and *yjbI*::Tn (Figure 1(b,c)).

**Figure 2.**
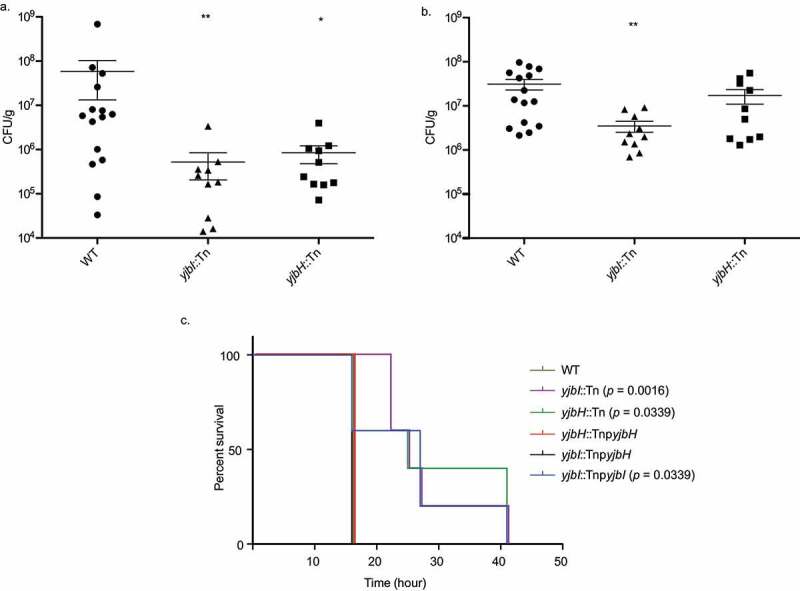
The *yjbH* gene is responsible for pathogenicity in mice. (a). The microbial burden in the heart. (b). The microbial burden in the kidney. Mice were injected intravenously with the strains into the tail vein. Bacteria recovered at 24 h post-infection from the isolated organs (kidney and heart) of the infected mice were counted for each strain. Each symbol represents data obtained from one animal. Data are shown as mean ± SEM of three experiments and analyzed by the Mann–Whitney U-test and significant differences compared to the wild-type are indicated by asterisks (*p ≤ 0.05, ** *p* ≤ 0.01). Injected CFU: WT: 8.5 × 10^7^–1.5x10^8^; *yjbI*::Tn: 1.58 × 10^8^–2.03x10^8^; *yjbH*::Tn: 1.05 × 10^8^–1.73x10^8^. (c). Survival of mice after systemic infection. Mice (n = 5) were injected intravenously with the strains into the tail-vein, and survival was observed. The Mantel-Cox log-rank test was used to compare the survival percentage of mice in different groups. Injected CFU: WT: 5.04 x10^8^; *yjbI*::Tn: 4.0 x10^8^, *yjbH*::Tn: 4.22 x10^8^, *yjbH*::Tnp*yjbH*: 5.02 x10^8^, *yjbI*::Tnp*yjbH*: 4.71 x10^8^, *yjbI*::Tnp*yjbI*: 6.95 × 10^8^

We next evaluated the pathogenicity of the mutants by determining the microbial burden in the organs of infected mice. We found that colonization of *yjbI*::Tn was reduced in kidney and heart [14] while that of *yjbH*::Tn was reduced only in the heart of infected mice (Figure 2(a,b)). To further confirm the killing ability of these strains, we examined the survival of mice in a systemic infection model. We found that the injection of wild-type, *yjbH*::Tnp*yjbH* and *yjbI*::Tnp*yjbH* strains killed all the mice within 16 hours, and the killing ability of *yjbI*::Tn, *yjbH*::Tn, and *yjbI*::Tnp*yjbI* strains was significantly decreased (Figure 2(c)). Thus, similar to the silkworm survival assay, mouse survival assay revealed that the YjbH was required for the killing ability of *S. aureus*.

### Protease and pigment production

**Figure 3.**
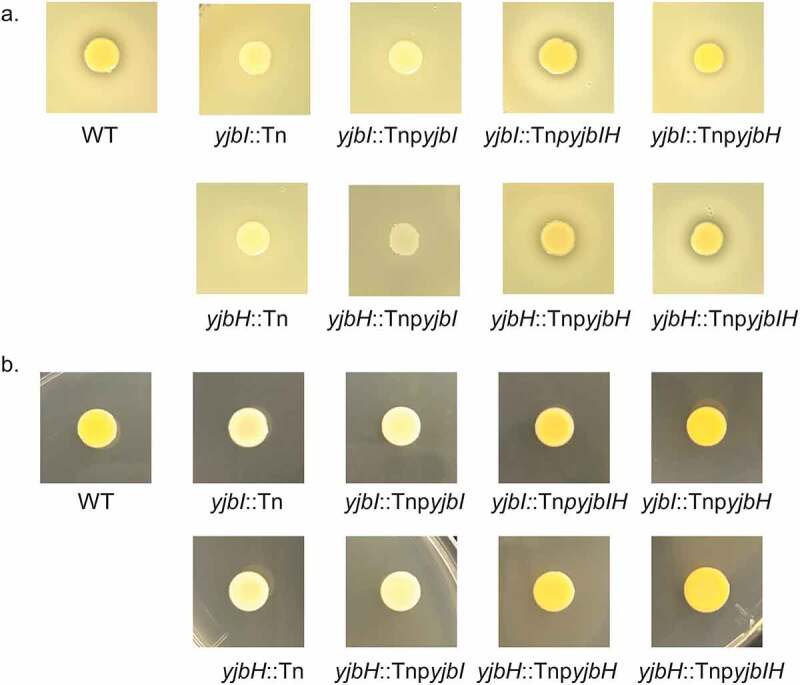
The *yjbH* gene is responsible for protease and pigment production. (a). Protease production. Two microliters of the overnight cultures of the strains were spotted on TSB+ 3.3% skim milk agar plates and incubated at 37°C overnight. (b). Pigment production. Two microliters of the overnight cultures of the strains were spotted on TSB agar plates and incubated at 37°C overnight

Our previous result [14] and other reports [20,32] demonstrated a reduction in protease and pigment production in the *yjbI* and *yjbH* gene-disrupted mutants. We obtained consistent results in this study (Figure 3(a,b)), and the protease and pigment production were restored by complementation of *yjbI*::Tn and *yjbH*::Tn with the *yjbIH* operon or the *yjbH* gene alone, but not with the *yjbI* gene (Figure 3(a,b)).

### *Surface protein and cell envelope glycopolymer structure were altered by disruption of the* yjbI *and the* yjbH *genes*

**Figure 4.**
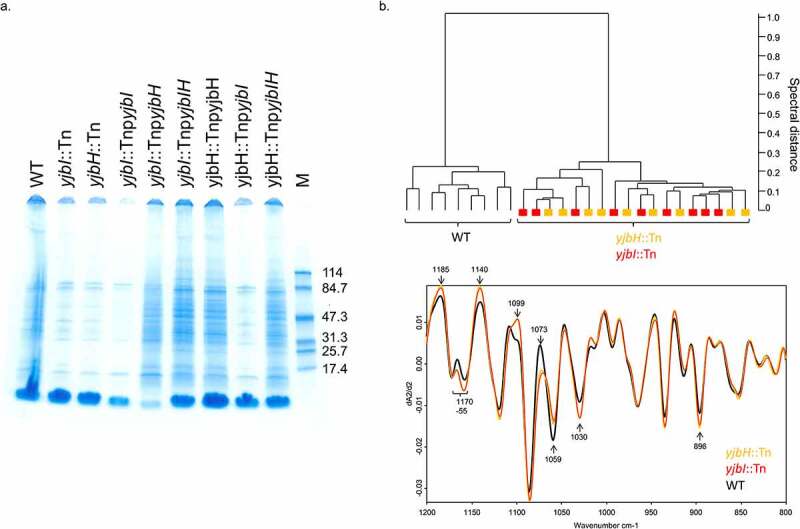
Alteration in surface structure due to the *yjbI* and the *yjbH* genes disruption. (a). Surface protein profiles of the strains. Surface proteins were extracted from the respective strains and applied to a 4–20% gradient SDS-PAGE gel and visualized by CBB staining. M: Molecular weight marker. (b). FTIR spectral profiling of cell envelope glycostructures. FTIR spectroscopy-based dendrogram (HCA) of wild-type, *yjbH*::Tn, and *yjbI*::Tn, each comprising triplicate measurements at three different days (upper). Average spectra were generated from the second derivative, vector-normalized FTIR spectra. Black: wild-type, orange: *yjbH*::Tn, red: *yjbI*::Tn (lower)

To get insight into the mechanism of reduced virulence by YjbH, we sought to analyze the surface protein profiles. We found that the *yjbH* and the *yjbI* genes-disrupted mutants had reduced surface proteins compared to that of the wild-type, which was complemented by the introduction of the mutants with the *yjbIH* operon and the *yjbH* gene but not the *yjbI* gene (Figure 4(a)). Next, we performed spectroscopic fingerprinting using Fourier-transform infrared (FTIR) spectroscopy for the *yjbI*::Tn, *yjbH*::Tn, and the wild-type strains to investigate changes in the cell envelope glycopolymer structure. We found that both the *yjbI*::Tn and *yjbH*::Tn mutants clustered distinct from the wild-type, but they could not be discriminated from each other using the highly discriminatory polysaccharide spectral region (Figure 4(b), upper). Disruption of the *yjbI* and the *yjbH* genes caused a large number of prominent spectral differences at wavenumbers between 1190–890 cm^−1^, which can be assigned to strong perturbations in the bacterial surface/cell wall-glycopolymer composition (Figure 4(b), lower). Here, the absence of YjbH led to an alteration in surface protein and cell envelope glycopolymer composition that could likely contribute to the virulence of *S. aureus* [33–35].

### RNA-seq analysis reveals downregulation of virulence and oxidative stress-related genes

**Figure 5.**
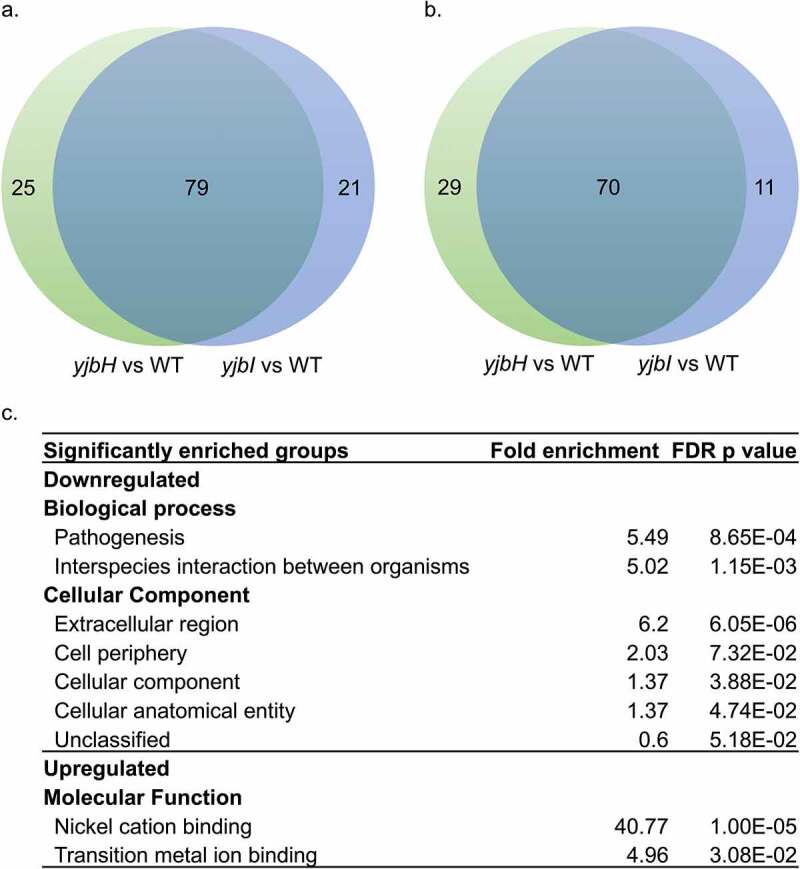
RNA-seq analysis showing the differentially expressed genes in *yjbH*::Tn and *yjbI*::Tn compared to that in the wild-type. (a). Genes that were significantly downregulated (<2-fold, FDR *p-value* <0.05). (b). Genes that were significantly upregulated (>2-fold, FDR *p-value* <0.05). (c). Gene Ontology term enrichment analysis. A complete list of differentially expressed genes in *yjbI*::Tn and *yjbH*::Tn compared with that of the wild-type (2 fold, FDR *p-value* <0.05) is shown in Supplemental Table S1 and S2

With the involvement of the *yjbI* and the *yjbH* genes in protease and pigment production, surface protein production and pathogenicity of *S. aureus*, we speculated YjbH might be involved in regulating the expression of various genes. Therefore, we performed RNA-seq analysis. We found that disruption of both the genes led to significant changes in the expression of the genes from diverse pathways. We found similar expression patterns among the *yjbI* and *yjbH* genes- disrupted mutants with a common upregulation and downregulation of 70 and 79 genes, respectively. (Figure 5(a,b), Supplementary Table S1, S2).

To gain functional insight into the differentially expressed genes common in both the gene disrupted mutants, we used Gene Ontology term enrichment analysis. We found that the genes involved in pathogenesis and extracellular region were highly represented among the downregulated genes. Some of the most downregulated genes included the SAUSA300_0113 (*spa)* gene, encoding the immunoglobulin G binding protein A precursor; SAUSA300_2453, encoding ATP-binding protein of ABC transporters; SAUSA300_2454, encoding a putative membrane-spanning protein; SAUSA300_0114 (*sarS*), and SAUSA300_1890, encoding staphopain A protease. In addition, the alternative sigma factor *sigB* and the genes whose transcription exclusively depends on SigB, such as *asp23*, SAUSA300_2143, and SAUSA300_2144, were downregulated (Supplementary Table S1), suggesting that YjbH contributes to the control of SigB activity. Further, expression of the *spxA* was downregulated, which might be due to the autoregulation of *spx* transcription due to increased Spx level [36]. We further observed a significant downregulation of zinc metalloprotease *aur* and other proteases such as serine and cysteine proteases, which explains the reduced protease production in these mutants.

Genes related to nickel cation binding were highly represented among the upregulated genes (Figure 5(c)). Along with others, this category included the genes involved in urease operon. Urease is essential for maintaining pH homeostasis in bacteria and upregulated in response to environmental changes, including acidic stress and urea and nitrogen depletion [37]. Since urease is a nickel metalloenzyme [38], nickel import is essential for urease function. Consistent with this, *nixA* encoding a nickel transporter, a putative urea transporter SAUSA300_2237, and SAUSA300_0231, encoding ABC transporter involved in nickel transport, were highly upregulated (Supplementary Table S2). Thus, YjbH might be necessary for pH homeostasis during acidic stress in *S. aureus*. These results suggested that YjbH is responsible for pleiotropic phenotypes in *S. aureus*, possibly by directly or indirectly regulating the expression of genes from diverse pathways and by the accumulation of Spx.

### Virulence is conferred by protection against oxidative stress

**Figure 6.**
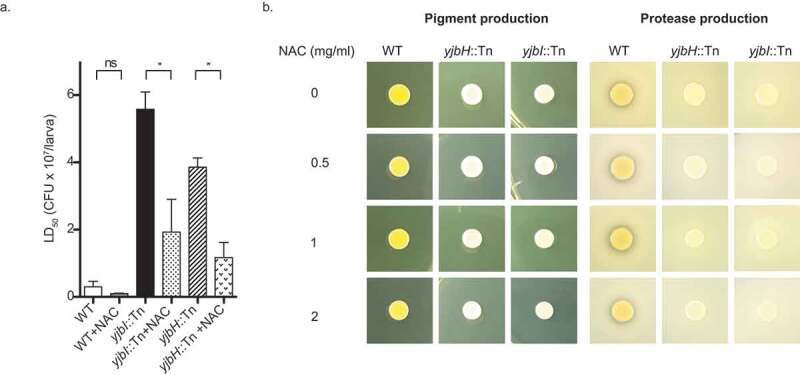
Effect of NAC upon pathogenicity, pigment production and proteolysis. (a). Pathogenicity of wild-type and mutants in the presence and absence of a free-radical scavenger. Silkworms were injected with NAC (2 mg/larva) before injecting with the different doses of the respective *S. aureus* strains, and survival of the silkworms was observed. The lethal dose fifty values were calculated at 30 h post-infection for all the strains in the presence and absence of NAC. Statistical analysis was performed by one-way ANOVA using Dunnett’s multiple comparison test. A *p-value* less than 0.05 was considered significant and indicated by an asterisk, ns: not significant. (b). Effect of NAC on pigment and protease production in vitro. Bacteria were grown in the presence of NAC at the concentrations indicated, and 2 µl of the overnight culture was spotted on either TSB or TSB +3.3% skim milk agar plate for pigment and protease production, respectively followed by overnight incubation

Previous reports have shown that YjbH is involved in controlling the protein levels of Spx, which is activated in response to disulfide and oxidative stress in *S. aureus* [15,16,18,20,25]. In our study, we observed the downregulation of several genes related to oxidative stress from the RNA-seq analysis (Supplementary Table S1). Thus, we were intrigued to test whether YjbH is involved in virulence by protecting *S. aureus* against oxidative stress in the host. For this purpose, we determined the lethal dose fifty (LD_50_) values of the strains in the presence and absence of N-acetyl-L-cysteine (NAC). NAC is a free radical scavenger; it eliminates reactive oxygen species (ROS) such as OH•, HOX, NO_2_, and H_2_O_2_ [39]. We found that by the pre-injection of NAC into silkworm hemolymph, LD_50_ values were significantly decreased, rendering the strains more effective in killing the silkworms (Figure 6(a)). We further found that NAC did not affect protease and pigment production by the mutants in vitro (Figure 6(b)). These results suggested that YjbH functions in protecting *S. aureus* from oxidative stresses in the host.

## Discussion

Our result is the first report showing the role of YjbH as a virulence factor of *S. aureus* in animal models, both silkworms and mice, and the first to show the function of YjbH regarding virulence is related to oxidative stress in the host. Previous studies had determined the viability of the *yjbH* and the *yjbI* genes-disrupted mutants in organs of infected mice [14,20]; however, our microbial viability data were different from the previous report [20]. In this study, we evaluated the killing ability of the mutants in mice systemic infection model and found that under our conditions, the mutants were less virulent. Moreover, transcriptome analysis suggested that YjbH acts as a regulator of expression of multiple genes and many of which were related to virulence and oxidative stress. Furthermore, YjbH seems to be necessary for the expression of various proteins located on the *S. aureus* surface and proteases.

Microbes acquire nitrogen through the degradation of proteins using proteases. Therefore, it is expected that the reduced protease production by the disruption of the *yjbH* gene resulted in insufficient nitrogen supply. As *S. aureus* can utilize urea as a nitrogen source [40], it is likely that *yjbH*::Tn enhanced urease production to compensate for the nitrogen supply in the absence of YjbH. This notion is further supported by the enhanced production of urease in the mutants with reduced proteolytic activities [41–43]. In addition to the genes involved in urease production, we found that the genes related to transport of nickel, a urease cofactor, were highly upregulated in the absence of YjbH. Given that urease is responsible for the conversion of urea into ammonia [44], which is an important pathway to alleviate acidic stress in many bacteria [45], YjbH might have a role in acidic stress resistance and pH homeostasis.

Staphyloxanthin, the yellow pigment, has been shown to have roles in protecting *S. aureus* against reactive oxygen species in the host [46]. The loss of yellow pigment in the absence of the *yjbH* gene suggests that YjbH exerts oxidative stress resistance by regulating staphyloxanthin production. Based on the effect of YjbH on other oxidative stress-related genes, it is evident that YjbH plays a role in providing oxidative stress tolerance to *S. aureus*, which is an important defense mechanism against host immunity. This function was further verified by our in vivo oxidative stress model using silkworm, where an ROS scavenger was able to rescue *S. aureus* against host-immunity and increased the virulence of *yjbH*::Tn.

In our RNA seq analysis, we found that the expression of stress regulators *spxA* and *sigB*, including SigB-specific genes, were downregulated. YjbH is involved in the proteolysis of Spx, a stress regulator required for oxidative stress tolerance [18,19,21,36]. In the absence of YjbH, Spx is accumulated, which represses its own expression [36]. The other stress regulator, SigB, is known to regulate a wide range of genes including many virulence factors [47]. Despite its ability to regulate virulence-related genes, the in vivo role of SigB in pathogenicity is disease model specific. For instance, *sigB* gene-deficient mutant was found to be less virulent in mouse models of arthritis [48] and intraperitoneal infection [49]; whereas mouse model of the abscess and hematogenous pyelonephritis [50], and a rat model of osteomyelitis [51] found no role of SigB in virulence of *S*. *aureus*. Similarly, the absence of SigB causes reduced pigmentation irrespective of the presence of YjbH or Spx, and the absence of Spx was required for the enhanced proteolytic activity of *sigB-yjbH* double gene disruptant [20]. Therefore, regulatory cross talks might exist between the stress regulators such as Spx, SigB, and YjbH, a detailed elucidation of which is further required.

In our study, we have shown for the first time that YjbH is required for full virulence of *S. aureus* and regulates virulence genes and surface proteins, and is necessary for oxidative stress tolerance inside the host. Moreover, the disruption of the *yjbI* and the *yjbH* genes led to reduced protease activity and reduced surface protein. Interestingly, most of the proteases are excreted into supernatant from the cell in general [52–54], and surface proteins of *S. aureus* predominantly consist of proteins related to adhesion and evasion such as clumping factor, fibronectin, and collagen-binding proteins, staphylococcal protein A [55–58]. In this regard, further proteomic analysis of the whole proteome, surfacome, and secretome of the mutants is required to provide an in-depth understanding of the role of YjbH in Staphylococcal virulence. Nonetheless, the findings of this study indicate that unlike other virulence factors such as *sarA, agr*, and other two-component systems such as *srrAB, saeRS*, and *ArlRS* [2], YjbH affects the expression of a broad range of genes. Further studies regarding the detailed mechanism at the protein level will give an insight into the complex regulatory mechanism of *S. aureus* virulence pathway and its utilization as a target for anti-virulence drug development.

## Supplementary Material

Supplemental Material
