# Supplementary material for: YjbH regulates virulence genes expression and oxidative stress resistance in Staphylococcus aureus
Source: Virulence. 2021 Jan 25;12(1):470–80. doi: 10.1080/21505594.2021.1875683 (PMC7849776; doi:10.1080/21505594.2021.1875683)
Supplement: Supplemental Material [file KVIR_A_1875683_SM4135.pdf]

Table S1 (Related to Figure 5). Complete list of genes downregulated as compared to wild type **in both *yjbH* and *yjbI***

Locus tag	Product	yjbH vs WT		yjbI vs WT	
		Fold	FDR p	Fold	FDR p
SAUSA300_0007	NAD(P)H-hydrate dehydratase	-2.98	0.00	-2.98	0.00
SAUSA300_0025	cyclic-nucleotide-phosphodiesterase	-5.65	0.00	-5.53	0.00
SAUSA300_0089	tRNA-dihydrouridine synthase	-2.11	0.04	-2.16	0.04
SAUSA300_0097	ATPase	-2.14	0.01	-2.06	0.03
SAUSA300_0099	1-phosphatidylinositol phosphodiesterase	-9.32	0.00	-8.88	0.00
SAUSA300_0112	L-lactate permease	-3.03	0.00	-2.94	0.00
SAUSA300_0113	peptidoglycan-binding protein LysM	-252.33	0.00	-235.42	0.00
SAUSA300_0114	transcriptional regulator	-12.91	0.00	-12.68	0.00
SAUSA300_0116	iron ABC transporter permease	-2.51	0.02	-2.48	0.04
SAUSA300_0117	iron ABC transporter substrate-binding protein	-4.72	0.00	-4.14	0.00
SAUSA300_0135	Superoxide dismutase [Mn/Fe] 2	-5.56	0.00	-4.99	0.00
SAUSA300_0136	hypothetical protein	-3.09	0.00	-3.52	0.00
SAUSA300_0188	branched-chain amino acid transporter II carrier	-3.67	0.00	-3.47	0.00
SAUSA300_0191	PTS glucose EIICBA component	-3.01	0.00	-3.13	0.00
SAUSA300_0230	PrsW family intramembrane metalloprotease	-2.14	0.01	-2.11	0.01
SAUSA300_0271	ABC transporter ATP-binding protein	-5.12	0.00	-3.49	0.01
SAUSA300_0272	ABC transporter permease	-6.05	0.00	-3.14	0.03
SAUSA300_0273	ABC transporter permease	-5.26	0.00	-7.92	0.00
SAUSA300_0274	CHAP domain-containing protein	-3.10	0.00	-2.43	0.03
SAUSA300_0277	virulence factor EsxA	-4.16	0.00	-3.46	0.00
SAUSA300_0278	protein EsaA	-2.99	0.00	-2.66	0.00
SAUSA300_0279	5'-nucleotidase, lipoprotein e(P4) family	-8.42	0.00	-8.32	0.00
SAUSA300_0307	lipase	-4.16	0.00	-4.16	0.00
SAUSA300_0320	glycerol-3-phosphate transporter	-2.52	0.01	-2.64	0.01
SAUSA300_0337	peptidase	-5.91	0.00	-4.61	0.00
SAUSA300_0372	GlsB/YeaQ/YmgE family protein	-2.26	0.03	-2.49	0.01
SAUSA300_0374	hypothetical protein	-4.75	0.01	-5.36	0.01
SAUSA300_0407	hypothetical protein	-2.86	0.01	-3.03	0.00
SAUSA300_0408	APC family permease	-2.35	0.00	-2.34	0.00
SAUSA300_0566	ABC transporter substrate-binding protein	-2.46	0.00	-2.37	0.00
SAUSA300_0598	alpha/beta hydrolase	-3.83	0.00	-3.75	0.00
SAUSA300_0604	MarR family transcriptional regulator	-2.47	0.00	-2.23	0.00
SAUSA300_0672	hypothetical protein	-2.68	0.05	-4.40	0.00
SAUSA300_0767	DUF5067 domain-containing protein	-5.75	0.00	-5.15	0.00
SAUSA300_0769	thermonuclease	-9.75	0.00	-10.97	0.00
SAUSA300_0776	CsbD family protein	-2.38	0.03	-2.39	0.03
SAUSA300_0816	regulatory protein Spx	-2.11	0.02	-2.00	0.04
SAUSA300_0898	glycosyl transferase family 1	-3.16	0.00	-2.86	0.00
SAUSA300_0939	staphopain B	-2.90	0.03	-2.99	0.03
SAUSA300_0950	serine protease	-4.07	0.00	-4.56	0.00
SAUSA300_0951	DUF5011 domain-containing protein	-2.03	0.03	-2.45	0.00
SAUSA300_0964	pyruvate carboxylase	-2.09	0.03	-2.12	0.03
SAUSA300_1014	succinate dehydrogenase flavoprotein subunit	-2.16	0.00	-2.16	0.00
SAUSA300_1047	tRNA (guanosine(37)-N1)-methyltransferase TrmD	-2.71	0.03	-3.03	0.01
SAUSA300_1133	PepSY domain-containing protein	-2.95	0.00	-3.23	0.00
SAUSA300_1334	monofunctional glycosyltransferase	-2.17	0.01	-2.05	0.03
SAUSA300_1855	YihY/virulence factor BrkB family protein	-2.60	0.00	-2.64	0.00
SAUSA300_1864	non-heme ferritin	-2.00	0.02	-2.40	0.00
SAUSA300_1874	sodium:proline symporter	-2.52	0.01	-2.47	0.01
SAUSA300_1883	staphopain A	-11.09	0.00	-11.46	0.00
SAUSA300_1890	chemotaxis inhibitory protein	-3.22	0.01	-3.43	0.01
SAUSA300_1920	staphylokinase	-6.69	0.00	-7.53	0.00
SAUSA300_1922	peptide chain release factor 1	-2.09	0.01	-2.09	0.02
SAUSA300_2072	NAD(P)-dependent oxidoreductase	-2.78	0.00	-2.29	0.01
SAUSA300_2097	Asp23/Gls24 family envelope stress response protein	-4.43	0.00	-3.44	0.00
SAUSA300_2142	DUF2273 domain-containing protein	-2.84	0.00	-2.68	0.00
SAUSA300_2143	hypothetical protein	-3.43	0.00	-3.62	0.00
SAUSA300_2144	hyaluronate lyase	-3.07	0.00	-3.01	0.00
SAUSA300_2161	AraC family transcriptional regulator	-5.41	0.00	-3.46	0.00
SAUSA300_2248	DUF1641 domain-containing protein	-2.88	0.00	-2.75	0.00
SAUSA300_2257	formate dehydrogenase subunit alpha	-2.51	0.00	-2.68	0.00
SAUSA300_2258	sodium ABC transporter ATP-binding protein	-2.64	0.02	-2.36	0.05
SAUSA300_2288	DUF805 domain-containing protein	-2.38	0.02	-2.93	0.00
SAUSA300_2289	sodium/glutamate symporter	-2.82	0.01	-3.08	0.00
SAUSA300_2291	immunoglobulin-binding protein sbi	-4.89	0.00	-4.53	0.00

SAUSA300_2364	ABC transporter ATP-binding protein	-2.08	0.03	-2.21	0.02
SAUSA300_2407	nickel ABC transporter	-2.25	0.01	-2.23	0.02
SAUSA300_2411	MFS transporter	-3.14	0.00	-2.92	0.00
SAUSA300_2449	lantibiotic ABC transporter ATP-binding protein	-170.64	0.00	-96.78	0.00
SAUSA300_2453	ABC transporter permease	-42.79	0.00	-45.04	0.00
SAUSA300_2454	N-acetyltransferase	-2.41	0.03	-3.08	0.00
SAUSA300_2468	PTS glucoside EIICBA component	-2.18	0.00	-2.18	0.00
SAUSA300_2476	4,4'-diaponeurosporenoate glycosyltransferase	-2.88	0.01	-2.54	0.03
SAUSA300_2500	diapolycopene oxygenase	-3.55	0.00	-3.08	0.00
SAUSA300_2501	alpha/beta hydrolase	-2.30	0.01	-2.06	0.03
SAUSA300_2518	hypothetical protein	-4.92	0.00	-4.20	0.00
SAUSA300_2528	citrate transporter	-2.31	0.01	-2.29	0.02
SAUSA300_2552	peptidase M4 family protein	-8.83	0.00	-11.49	0.00
SAUSA300_2572		-9.87	0.00	-11.58	0.00
***yjbH*-specific**					
SAUSA300_0196	type-1 restriction enzyme R protein	-2.03	0.00	-1.98	0.00
SAUSA300_0208	sugar ABC transporter ATP-binding protein	-4.52	0.00	-2.81	0.07
SAUSA300_0209	maltose ABC transporter substrate-binding protein	-4.18	0.01	-2.26	0.21
SAUSA300_0215	membrane protein	-2.74	0.01	-2.26	0.06
SAUSA300_0285	virulence factor EsxB	-3.75	0.01	-1.97	0.29
SAUSA300_0286	DUF5081 domain-containing protein	-3.49	0.03	-2.32	0.21
SAUSA300_0287	hypothetical protein	-4.05	0.03	-3.01	0.12
SAUSA300_0435	ABC transporter ATP-binding protein	-4.95	0.04	-3.88	0.12
SAUSA300_0505	pyridoxal 5'-phosphate synthase glutaminase subunit PdxT	-2.04	0.02	-1.89	0.04
SAUSA300_0506	pyrimidine nucleoside transporter NupC	-2.24	0.00	-1.96	0.00
SAUSA300_0773	coagulase	-2.95	0.04	-2.08	0.24
SAUSA300_0786	organic hydroperoxide reductase OsmC/OhrA	-3.25	0.04	-1.35	0.74
SAUSA300_1029	iron-regulated surface determinant protein A	-2.33	0.01	-1.56	0.26
SAUSA300_1136	ribosome biogenesis GTPase YlqF	-2.14	0.00	-1.79	0.05
SAUSA300_1222	thermonuclease	-3.53	0.02	-2.57	0.12
SAUSA300_1591	adenine phosphoribosyltransferase	-2.07	0.01	-1.79	0.07
SAUSA300_1683	bifunctional 3-deoxy-7-phosphoheptulonate synthase/chorismate mutase	-2.04	0.03	-1.88	0.07
SAUSA300_1807	amino acid ABC transporter ATP-binding protein	-2.85	0.02	-1.87	0.24
SAUSA300_2022	RNA polymerase sigma factor SigB	-2.21	0.00	-1.93	0.00
SAUSA300_2324	PTS sucrose transporter subunit IIBC	-2.10	0.01	-1.82	0.08
SAUSA300_2331	transcriptional regulator	-2.21	0.01	-1.96	0.03
SAUSA300_2351	zinc ABC transporter substrate-binding protein	-2.19	0.00	-1.86	0.02
SAUSA300_2524	TIGR04197 family type VII secretion effector	-3.09	0.05	-2.00	0.30
SAUSA300_2525	fructosamine kinase family protein	-2.15	0.03	-1.79	0.16
SAUSA300_2627	anion permease	-2.05	0.01	-1.83	0.03
***yjbI* -specific**					
SAUSA300_0147	bifunctional metallophosphatase/5'-nucleotidase	-1.67	0.18	-2.53	0.01
SAUSA300_0235	L-lactate dehydrogenase	-7.84	0.05	-9.65	0.03
SAUSA300_0292	hypothetical protein	-2.84	0.10	-4.13	0.03
SAUSA300_0557	HAD family hydrolase	-1.72	0.15	-2.42	0.01
SAUSA300_0669	undecaprenyl-diphosphatase	-1.88	0.02	-2.03	0.01
SAUSA300_0793	DUF1963 domain-containing protein	-1.82	0.13	-2.38	0.02
SAUSA300_0903	hypothetical protein	-1.98	0.05	-91.59	0.00
SAUSA300_1054	hypothetical protein	-2.70	0.11	-3.59	0.04
SAUSA300_1137	ribonuclease HII	-1.66	0.14	-2.04	0.03
SAUSA300_1582	CsbD family protein	-2.45	0.06	-3.25	0.01
SAUSA300_2023	anti-sigma B factor RsbW	-1.98	0.03	-2.01	0.03
SAUSA300_2071	protein-(glutamine-N5) methyltransferase, release factor-specific	-1.79	0.11	-2.04	0.04
SAUSA300_2247	transcriptional regulator	-3.09	0.05	-4.37	0.02
SAUSA300_2286	membrane protein	-1.55	0.35	-2.34	0.03
SAUSA300_2287	ABC transporter permease	-1.93	0.05	-2.31	0.01
SAUSA300_2343	nitrate reductase subunit alpha	-3.14	0.26	-6.60	0.04
SAUSA300_2397	MFS transporter	-1.78	0.08	-2.16	0.01
SAUSA300_2408	ABC transporter ATP-binding protein	-2.23	0.07	-3.03	0.01
SAUSA300_2416		-1.82	0.18	-2.56	0.03
SAUSA300_2435	accumulation-associated protein	-1.36	0.86	-7.69	0.03
SAUSA300_2626	DinB family protein	-1.72	0.33	-3.06	0.03

Table S2 (Related to Figure 5). Complete list of genes upregulated as compared to wild type **in both *yjbH* and *yjbI***

Locus tag	Product	yjbH vs WT		yjbI vs WT	
		Fold	FDR p	Fold	FDR p
SAUSA300_0030	nickel ABC transporter substrate-binding protein	4.08	0.00	4.14	0.00
SAUSA300_0078	glycyl-glycine endopeptidase LytM	3.20	0.00	3.04	0.00
SAUSA300_0079	cysteine synthase	2.61	0.00	2.31	0.00
SAUSA300_0231	cystathionine gamma-synthase	3.18	0.00	2.65	0.00
SAUSA300_0270	hypothetical protein	3.03	0.00	2.69	0.00
SAUSA300_0433	hypothetical protein	3.08	0.00	3.02	0.00
SAUSA300_0434	ABC transporter ATP-binding protein	2.31	0.00	2.30	0.00
SAUSA300_0442	DUF1361 domain-containing protein	2.75	0.01	2.41	0.04
SAUSA300_0443	thiol reductase thioredoxin	2.24	0.01	2.06	0.03
SAUSA300_0630	oligoendopeptidase F	2.52	0.00	2.66	0.00
SAUSA300_0678	acyltransferase	2.21	0.02	2.03	0.05
SAUSA300_0789	hypothetical protein	2.06	0.01	2.07	0.01
SAUSA300_0902	5-(carboxyamino)imidazole ribonucleotide synthase	2.66	0.02	2.76	0.01
SAUSA300_0953	cytochrome ubiquinol oxidase subunit I	3.51	0.00	3.57	0.00
SAUSA300_0957	non-canonical purine NTP pyrophosphatase	2.23	0.01	2.15	0.02
SAUSA300_0967	hemolytic protein	8.94	0.00	7.38	0.00
SAUSA300_0986	low specificity L-threonine aldolase	4.15	0.00	2.92	0.03
SAUSA300_1050	hypothetical protein	2.27	0.00	2.30	0.00
SAUSA300_1067	hypothetical protein	2.62	0.00	2.42	0.00
SAUSA300_1068	SWIM zinc finger family protein	2.31	0.01	2.59	0.00
SAUSA300_1214	hypothetical protein	2.83	0.03	3.22	0.01
SAUSA300_1223	acylphosphatase	3.85	0.00	4.17	0.00
SAUSA300_1248	5-bromo-4-chloroindolyl phosphate hydrolysis protein	2.79	0.00	3.42	0.00
SAUSA300_1272	tellurite resistance protein TelA	2.87	0.00	3.00	0.00
SAUSA300_1277	hypothetical protein	2.08	0.02	2.06	0.03
SAUSA300_1297	DUF1798 domain-containing protein	2.81	0.01	2.82	0.01
SAUSA300_1298	30S ribosomal protein S1	2.03	0.02	2.00	0.03
SAUSA300_1299	dipeptidase	2.03	0.02	2.18	0.01
SAUSA300_1338	hypothetical protein	2.57	0.04	2.91	0.02
SAUSA300_1339	multidrug ABC transporter ATP-binding protein	2.65	0.00	2.54	0.00
SAUSA300_1365	ABC transporter ATP-binding protein	2.62	0.00	2.54	0.00
SAUSA300_1654	aldehyde dehydrogenase	2.17	0.01	2.18	0.01
SAUSA300_1721	ABC transporter ATP-binding protein	2.09	0.00	2.02	0.00
SAUSA300_1747	purine-nucleoside phosphorylase	2.05	0.00	2.09	0.00
SAUSA300_1785	ISL3 family transposase	25.51	0.00	12.01	0.02
SAUSA300_1786	aldo/keto reductase	3.20	0.01	3.08	0.02
SAUSA300_1901	peptidase M23	2.14	0.04	2.23	0.04
SAUSA300_1913	AcrB/AcrD/AcrF family protein	2.20	0.00	2.19	0.00
SAUSA300_2091	urea transporter	6.65	0.00	7.67	0.00
SAUSA300_2115	urease subunit gamma	14.13	0.00	10.97	0.00
SAUSA300_2159	urease subunit beta	13.94	0.00	11.00	0.00
SAUSA300_2162	urease subunit alpha	9.99	0.00	8.97	0.00
SAUSA300_2213	urease accessory protein UreE	13.50	0.00	12.43	0.00
SAUSA300_2237	urease accessory protein UreF	10.53	0.00	9.79	0.00
SAUSA300_2238	urease accessory protein UreG	7.31	0.00	5.44	0.00
SAUSA300_2239	urease accessory protein	4.63	0.00	3.99	0.00
SAUSA300_2240	CHAP domain-containing protein	2.95	0.00	2.70	0.00
SAUSA300_2241	CPBP family intramembrane metalloprotease	2.20	0.00	2.21	0.00
SAUSA300_2242	3-hydroxyacyl-CoA dehydrogenase	2.44	0.05	2.57	0.04
SAUSA300_2243	N-acetyltransferase	5.49	0.00	5.06	0.00
SAUSA300_2244	ferredoxin--NADP(+) reductase	2.66	0.00	2.81	0.00
SAUSA300_2253	amino acid ABC transporter permease	3.51	0.02	3.18	0.03
SAUSA300_2262	amino acid transporter	3.35	0.00	2.82	0.01
SAUSA300_2294	glycine/betaine ABC transporter ATP-binding protein	5.25	0.00	5.17	0.00

SAUSA300_2318		2.22	0.02	2.10	0.03
SAUSA300_2319		2.22	0.02	2.10	0.03
SAUSA300_2390		4.77	0.00	4.62	0.00
SAUSA300_2391	GTP pyrophosphokinase	2.00	0.03	2.30	0.01
SAUSA300_2393	DedA family protein	3.10	0.00	3.29	0.00
SAUSA300_2440	NAD(P)H-dependent oxidoreductase	2.84	0.00	2.72	0.00
SAUSA300_2440	acyl-CoA thioester hydrolase	2.49	0.03	2.51	0.03
SAUSA300_2441	copper-exporting P-type ATPase A	2.32	0.00	2.10	0.01
SAUSA300_2446	VOC family protein	2.66	0.00	2.81	0.00
SAUSA300_2450	class I fructose-bisphosphate aldolase	2.13	0.01	2.20	0.01
SAUSA300_2462	HoxN/HupN/NixA family nickel/cobalt transporter	3.58	0.00	3.67	0.00
SAUSA300_2475		2.33	0.00	2.26	0.00
SAUSA300_2494		7.50	0.00	7.26	0.00
SAUSA300_2529		2.79	0.03	2.85	0.03
SAUSA300_2540		2.02	0.02	2.17	0.01
SAUSA300_2630		2.90	0.01	2.55	0.03
***yjbH*-specific**					
SAUSA300_0248	CDP-glycerol--glycerophosphate glycerophosphotransferase	2.03	0.019	1.98	0.031
SAUSA300_0381	NADPH-dependent oxidoreductase	2.1	0.013	1.98	0.031
SAUSA300_0393	DUF1304 domain-containing protein	7.27	0.032	5.52	0.101
SAUSA300_0686	N-acetylglucosamine-6-phosphate deacetylase	2.04	0.014	1.69	0.12
SAUSA300_0850	Na+/H+ antiporter subunit F	2.12	0.037	1.82	0.153
SAUSA300_0958	hypothetical protein	2.05	0.001	1.94	0.005
SAUSA300_1020	glycerophosphodiester phosphodiesterase	2.03	0.031	1.84	0.088
SAUSA300_1049	glutamate racemase	2.12	0.03	1.98	0.063
SAUSA300_1273	ABC transporter ATP-binding protein	2.33	0.02	2.09	0.063
SAUSA300_1296	DUF1033 domain-containing protein	2.52	0.023	1.97	0.165
SAUSA300_1326	ribonuclease H	3.03	0.036	2.71	0.088
SAUSA300_1451	SDR family oxidoreductase	2.25	0.023	2.09	0.054
SAUSA300_1548	ComE operon protein 2	2.1	0.031	1.9	0.091
SAUSA300_1640	isocitrate dehydrogenase (NADP(+))	2.29	2E-05	1.99	1E-03
SAUSA300_1667	glycerophosphoryl diester phosphodiesterase	2.88	0.019	2.42	0.081
SAUSA300_1674	serine protease	2.08	0.001	1.96	0.004
SAUSA300_1939		2.41	0.048	2.4	0.058
SAUSA300_1988		2952	2E-04	-1.16	0.999
SAUSA300_2105	PTS mannitol transporter subunit IICB	2.13	0.031	1.58	0.313
SAUSA300_2206	hypothetical protein	2.41	0.013	1.81	0.168
SAUSA300_2295	hypothetical protein	2.19	0.015	1.78	0.137
SAUSA300_2300	TetR family transcriptional regulator	2.63	0.008	2.15	0.06
SAUSA300_2323	magnesium transporter CorA	2.02	0.014	1.95	0.029
SAUSA300_2392	choline ABC transporter permease	3.39	0.047	2.76	0.144
SAUSA300_2421	DUF1433 domain-containing protein	2.53	0.03	1.83	0.262
SAUSA300_2422	oxidoreductase	2.21	0.013	1.96	0.055
SAUSA300_2473	alpha/beta hydrolase	2.04	0.004	1.97	0.009
SAUSA300_2495	copper chaperone CopZ	2.79	0.008	2.23	0.061
SAUSA300_2559	DNA-binding response regulator	2.29	0.012	1.92	0.083
***yjbI* -specific**					
SAUSA300_0905	CYTH domain-containing protein	1.83	0.094	2.05	0.039
SAUSA300_0968	cardiolipin synthase	1.99	0.003	2.07	0.002
SAUSA300_0969	peroxiredoxin	1.95	0.047	2.47	0.003
SAUSA300_0987	ABC-2 transporter family protein	1.69	0.22	2.24	0.029
SAUSA300_1216	dUTP pyrophosphatase	1.52	0.642	3.35	0.038
SAUSA300_1844		2.47	0.182	3.42	0.042
SAUSA300_1912	sulfurtransferase FdhD	2.06	0.089	2.44	0.029
SAUSA300_1941		2.27	0.071	2.55	0.036
SAUSA300_1949		2.66	0.063	3.15	0.029
SAUSA300_2231	cytochrome D ubiquinol oxidase subunit II	2.22	0.063	3.16	0.003
SAUSA300_2353	phosphoribosylaminoimidazolesuccinocarboxamide synthase	2.62	0.059	3.08	0.025
